# Reorganization of cortical individualized differential structural covariance network is associated with regional morphometric changes in chronic subcortical stroke

**DOI:** 10.1016/j.nicl.2025.103735

**Published:** 2025-01-13

**Authors:** Hongchuan Zhang, Jun Guo, Jingchun Liu, Caihong Wang, Hao Ding, Tong Han, Jingliang Chen, Chunshui Yu, Wen Qin

**Affiliations:** a Department of Radiology, Tianjin Key Lab of Functional Imaging & Tianjin Institute of Radiology, Tianjin Medical University General Hospital, Tianjin 300052, China; b Department of Radiology, Yijishan Hospital of Wannan Medical College, No.2 Zheshan West Road, Wuhu 241001, China; c Department of Radiology, Tianjin Huanhu Hospital, Tianjin 300350, China; d Tianjin University Huanhu Hospital, Tianjin 300350, China; e Department of Magnetic Resonance Imaging, The First Affiliated Hospital of Zhengzhou University, Zhengzhou 450052, China; f School of Medical Imaging, Tianjin Medical University, Tianjin 300070, China; g State Key Laboratory of Experimental Hematology, Tianjin 300070, China

**Keywords:** Subcortical stroke, IDSCN, Network-based statistics, Sub-network

## Abstract

Patients with chronic subcortical stroke undergo regional and network morphometric reorganizations beyond the lesion site, but the interplay between network and regional reorganization remains poorly understood. We aimed to clarify the reorganization patterns of the individualized differential structural covariance networks (IDSCN) in chronic subcortical stroke and investigate their associations with regional gray matter volume (GMV) changes and functional recovery. Structural MRI from four datasets enrolled 112 patients with chronic subcortical stroke (81 male, age: 55.82 ± 7.79) and 122 matched healthy controls (HC) (74 male; age: 55.28 ± 7.54). Network-based statistics were employed to identify aberrant IDSCN, Spearman correlation was conducted to assess the association between IDSCN and regional GMV alterations, and partial correlation was utilized to investigate the association between abnormal IDSCN and functional recovery. We identified 133 connections with balanced increased and decreased IDSCN. Aberrant IDSCN involved more regions than local GMV alterations, local GMV alteration exhibited intricate correlations with IDSCN, which could explain partly IDSCN reorganization (p < 0.05, corrected). Finally, abnormal IDSCN showed a weak association with long-term clinical recovery (p < 0.01). These findings reinforce the theory of adaptive network reorganization post-stroke and suggest that IDSCN may provide further insights into cortical reorganization and functional rehabilitation beyond regional morphometric measures.

## Highlights

• Chronic subcortical stroke suffered both increase and decrease in cortical IDSCN.
• Disrupted IDSCN impacts interhemispheric/contralesional VAN/SN and ipsilesional SMN.
• IDSCN offers cortical reorganization for subcortical stroke beyond local GMV.

## 1 Introduction

Chronic subcortical stroke is a debilitating condition characterized by focal infarctions within the subcortical regions (Guggisberg et al., 2019). Traditionally, the focus has been on understanding the effects of lesions on the structure and function of specific brain areas (Lee et al., 2015, Crofts et al., 2020). However, recent studies have suggested that chronic subcortical infarction may also lead to widespread cortical network disruption which is associated with multidimensional functional deficits (Alexander-Bloch et al., 2013). Advanced magnetic resonance imaging (MRI) can be utilized to construct the brain large-scale networks in an in-vivo and non-invasive manner. These include functional networks derived from resting-state MRI (rs-MRI) (Alexander-Bloch et al., 2013); anatomical networks from diffusion MRI (dMRI) (Diao et al., 2017); and structural covariance network (SCN) from structural MRI (sMRI) (Wang et al., 2019). Among these brain network approaches, SCN measures the morphometric covariance (similarity) between pairs of brain regions (Evans, 2013). Several morphometric measures can be used to construct the SCN, including gray matter volume (GMV) (Yang et al., 2016); cortical surface area (Sanabria-Diaz et al., 2010); cortical gyrification (Chen et al., 2013); cortical thickness (Wang et al., 2023) and so on (Zheng et al., 2018). Due to the readily accessible and highly reliable nature of sMRI data, SCN has gained widespread popularity in the fields of neuroscience and neurology (Wagner et al., 2020, Wei et al., 2020, Michels et al., 2022, Cotton et al., 2020). Notably, SCN has demonstrated promising capabilities in detecting brain network disruptions following subcortical stroke (Wang et al., 2019, Chen et al., 2022).

It is generally acknowledged that subcortical stroke exhibits high inter-subject heterogeneities in lesion locations and extents (Wen et al., 2013); which may relate to varying patterns of network disruption and reorganization (Hong et al., 2020, Yang et al., 2015, Zhao et al., 2021) as well as clinical outcomes (Yang et al., 2015, Bamiou, 2015, Kim et al., 2021). However, the predominant studies have adopted a group-wise SCN method by measuring the morphometric covariance between brain regions across subjects, which fails to capture the personalized network variability. To solve the drawback of the group-wise SCNs, several individualized SCN methods emerged in recent years, such as morphometric similarity network (Vuksanovic, 2022); Kullback–Leibler divergence similarity-based morphological brain network (Kong et al., 2015); individualized differential structural covariance network (IDSCN) (Liu et al., 2021); and so on. As a novel individualized SCN measure introduced by Liu et al at 2021 (Liu et al., 2021); IDSCN first constructs a reference group-wise SCN from a population (for example, all healthy controls [HC]), then derives a differential SCN of one subject by measuring the deviations of a perturbed SCN (by inserting this subject) relative the reference (Liu et al., 2021). This measure has been applied to various neuropsychiatric diseases and shows promise in addressing potential in resolving heterogeneity of brain impairment, such as schizophrenia (Liu et al., 2021), obsessive–compulsive disorder (OCD) (Han et al., 2022), and depressive disorder (Han et al., 2022).

The IDSCN has predominantly been utilized as a tool for delving into the nuanced and complex disruptions occurring within the cerebral architecture due to diffusely distributed brain pathologies (Conrad et al., 2022, Salvalaggio et al., 2020). However, the extent to which focal brain lesions—such as those caused by subcortical infarcts—impinge upon and perturb the SCN remains insufficiently understood. Building upon the foundational work that has illuminated the regional brain structural damage and the adaptive reorganization in distant brain regions after focal subcortical infarction (Wen et al., 2013, Salvalaggio et al., 2020); this study aims to expand a hypothesis that chronic subcortical infarctions are not merely isolated events with localized structural damage but can indeed provoke widespread disruptions of cortical IDSCN. This hypothesis is predicated on the notion that the structural integrity of the brain's network is intricately connected, such that a disturbance in one node invariably reverberates throughout the network (Veldsman et al., 2020, Nemati et al., 2022). Furthermore, we propose that these IDSCN disruptions could uncover the extent of heterogeneity in localized structural changes and functional deficits observed in chronic subcortical stroke.

## 2 Method and Materials

### 2.1 Participants

**Fig. 1:**
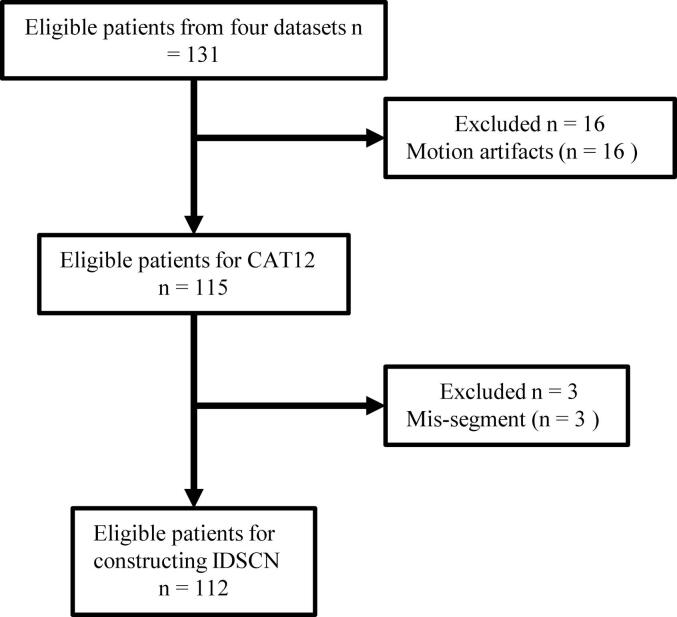
This chart illustrates the steps for image quality control in stroke patients. Abbreviations: IDSCN = individualized differential structural covariance network, CAT12 = Computational Anatomy Toolbox.

Four chronic subcortical stroke datasets were recruited from three centers: one dataset from the First Affiliated Hospital of Zhengzhou University, one from the Tianjin HuanHu Hospital, and two from Tianjin Medical University General Hospital by two scanners. The inclusion criteria for patients were as follows: (1) first onset ischemic stroke; (2) lesion located in the same hemisphere subcortex; (3) right-handed before the stroke; and (4) more than 6 months since stroke onset. The exclusion criteria for patients were: (1) recurrent stroke after first onset; (2) any other brain abnormalities; (3) a history of drug dependency or psychiatric disorders; (4) any contraindications to magnetic resonance examination; (5) more than one lesion within the brain. Finally, a total number of 112 patients ***(6, 7)*** (81 males, 40 ∼ 75 years old) were included according to the criteria after excluding images with motion artifacts and lesions located in the cortex Fig. 1. In addition, 122 HC (74 males, 40 ∼ 75 years old) were recruited under the same criteria, except for the absence of stroke episodes. Several dimensions of clinical assessments were applied to evaluate the functional status of subjects, including the Fugl-Meyer test (FMT), Flanker test, number 1-back test, and spatial 1-back test **(**Table 1**,** Supplementary Table S1**)**.

**Table 1:** Demographic and clinical information for participants.

	Stroke Patients (n = 112)	Healthy Control (n = 122)	Statistics	*p* values
Age (years)	55.82 ± 7.79	55.28 ± 7.54	T = 0.54	5.89e-01
Gender (M/F)	81/31	74/48	χ2 = 3.55	7.20e-02
Lesion (Left/Right)	57/55	/	/	/
Clinical Scores				
FMT	100(96,100)	100(100,100)	Z = −6.19	6.09e-10*
F_RT	547(500,774)	538(476,590)	Z = 1.81	6.40e-02
F_ACC	0.980(0.950,1.000)	0.983(0.970,1.000)	Z = −1.85	6.90e-02
N_RT	787(727,1018)	759(673,921)	Z = 1.73	8.30e-02
N_ACC	0.950(0.876,0.970)	0.930(0.884,0.955)	Z = 0.30	7.63e-01
S_RT	865(754,1036)	792(691,944)	Z = 2.42	1.50e-02*
S_ACC	0.920(0.867,0.950)	0.930(0.889,0.950)	Z = −1.52	1.27e-01

* Uncorrected p < 0.05. Abbreviations: FMT = Fugl-Meyer Test total scores; F_RT = Reaction Time of Flanker test; F_ACC = Accuracy of Flanker test; N_RT = Reaction Time of Number 1-back test; N_ACC = Accuracy of Number 1-back test; S_RT = Reaction Time of Spatial 1-back test; S_ACC = Accuracy of Spatial 1-back test.

All stroke patients and HC were provided with written informed consent before participating in this study. The ethics committee of Tianjin Medical University General Hospital, Tianjin Huanhu Hospital, and The First Affiliated Hospital of Zhengzhou University gave ethical approval for this work.

### 2.2 MR data acquisition and preprocessing

High-resolution 3D T1-weighted sMRI data were obtained using four 3.0-Tesla MR scanners from three hospitals. The detailed machine models and sequence acquisition parameters are shown in Supplementary Table S2.

### 2.3 Tissue segmentation and GMV extraction

CAT12 (https://neuro-jena.github.io/cat/) was used to preprocess the sMRI data with the following steps: (1) the brain was segmented into grey matter (GM), white matter (WM), and cerebral spinal fluid (CSF) after skull-stripping; (2) the GM map was normalized into MNI152 standard space using DARTEL algorithms, modulated with Jacobian determinants to obtain the absolute GMV, and resliced with a voxel size of an isotropic 1.5- mm; (3) the GMV map was smoothed using an isotropic Gaussian kernel (8 mm full wide at half maximum). Finally, we excluded voxels involved in infarct lesions and parceled the GMV of 218 cortical regions using the Brainnetome Atlas (BNA) (http://atlas.brainnetome.org/) (Fan et al., 2016).

### 2.4 IDSCN Construction

We constructed the IDSCN based on the pipeline outlined by Liu et al. (Liu et al., 2021) using the MATLAB 2016b package (https://www.mathworks.com/products/matlab.html) with the following steps: (1) for each dataset, a group SCN norm was built based on all HC participants of this dataset using partial correlation controlling for age, gender, and total intracranial volume (TIV), returning a normal SCN (nSCN) matrix of 218*218. The reason for creating separate norms for each dataset rather than a single norm for all datasets was to minimize the system bias on GMV and SCN quantification caused by different scanners (Man et al., 2021, Xu et al., 2020). (2) for each dataset, we added one patient ***k*** to the HC group to calculate the SCN again and get a perturbed SCN (pSCN); (3) we calculated the differential SCN between pSCN and nSCN (ΔSCN = pSCN − nSCN); (4) We calculated the Z score of ΔSCN (termed IDSCN) based on a pioneering work (Liu et al., 2016) demonstrating that ΔSCN follows a symmetrical “volcano” distribution with tail similar to normal distribution (Equation (1)):

(1) $$Z=\frac{\Delta SCN}{\frac{1-{\mathrm{nSCN}}^{2}}{n-1}}$$

In which n denotes the number of HC participants during the creation of the norms. Prior to statistical analysis, the IDSCNs of patients who had lesions on the right side were mirrored to establish a unified representation of the IDSCN patterns for the ipsilesional (same side as the lesion) and contralesional (opposite side of the lesion) hemispheres. The workflow of IDSCN calculation is illustrated in the Supplementary Fig. S2.

## 3 Statistical analyses

### 3.1 Demography and clinical statistics

The distributions of continuous data were evaluated using a one-sample Kolmogorov-Smirnov test. Inter-group differences in continuous variables were compared using either a two-sample *t*-test (for normal distribution) or the Mann-Whitney *U* test (for non-normal distribution). Categorical variables, including gender, were analyzed using the chi-square test (p < 0.05, uncorrected).

### 3.2 Network-Based Statistics of IDSCN

A nonparametric network-based statistics (NBS) was performed to identify average abnormal IDSCN connections in patients with subcortical stroke using GRETNA v2.0.0 (https://www.nitrc.org/projects/gretna/), with 1000 iterations, connection threshold of p < 0.001, and cluster threshold of family-wise error (FWE) corrected p < 0.05. We further categorized the identified abnormal IDSCN connections into eight subnets based on the YEO atlas (Yeo et al., 2011): Auditory Network (AN), Control Network (CN), Default Mode Network (DMN), Dorsal Attention Network (DAN), Limbic Network (LN), Somatic Motor Network (SMN), Ventral Attention Network (VAN) (which also include salience network [SN]), and Visual Network (VN). We summarized the connections and nodes with abnormal IDSCN by involved hemispheres and subnets, separately.

### 3.3 Regional GMV change and association with IDSCN

A two-sample *t*-test was used to compare the differences in cerebral regional GMV between stroke patients and HC (p < 0.05, FWE correction for effective number of tests) (Galwey, 2009). Subsequently, a Spearman correlation was used to assess the relationship between regional GMV changing and IDSCN across regions (p < 0.05, false discovery rate (FDR) correction).

### 3.4 Association between IDSCN and clinical measures

A partial correlation was applied to explore the associations between clinical measures and aberrant IDSCN connections, controlling for gender and age effects using FDR correction (p < 0.05).

The aforementioned statistics were conducted using Matlab 2016b and SPSS v19 (https://www.ibm.com/spss).

## 4 Results

### 4.1 Demography

As shown in Table 1, there were no significant differences in age (two-sample *t*-test, t = 0.54, p = 0.710) and gender (chi-square = 3.55, p = 0.072) between the chronic stroke patients and HC, which was replicated in each site (**Supplementary** Table S2). Most of the stroke patients achieved full recovery in motor function (FMT quantile = [96,100]). Besides, stroke patients exhibited a trend of longer reaction time during Flanker (Z = 1.81, p = 0.064), number 1-back (Z = 1.73, p = 0.083), and spatial 1-back tasks (Z = 2.42, p = 0.015), and poor Flanker accuracy (Z = −1.85, p = 0.069) compared to HC. At the site level, significantly lower FMT of stroke patients in the TJHH, FAHZU, and TMUGH1 sites (all p < 0.005), the longer reaction time of spatial 1-back task (p = 0.034) in TMUGH1, and lower accuracy of the spatial 1-back task in TJHH, were also observed (Supplementary Table S1**)**.

### 4.2 Global IDSCN aberrance in chronic stroke

**Fig. 2:**
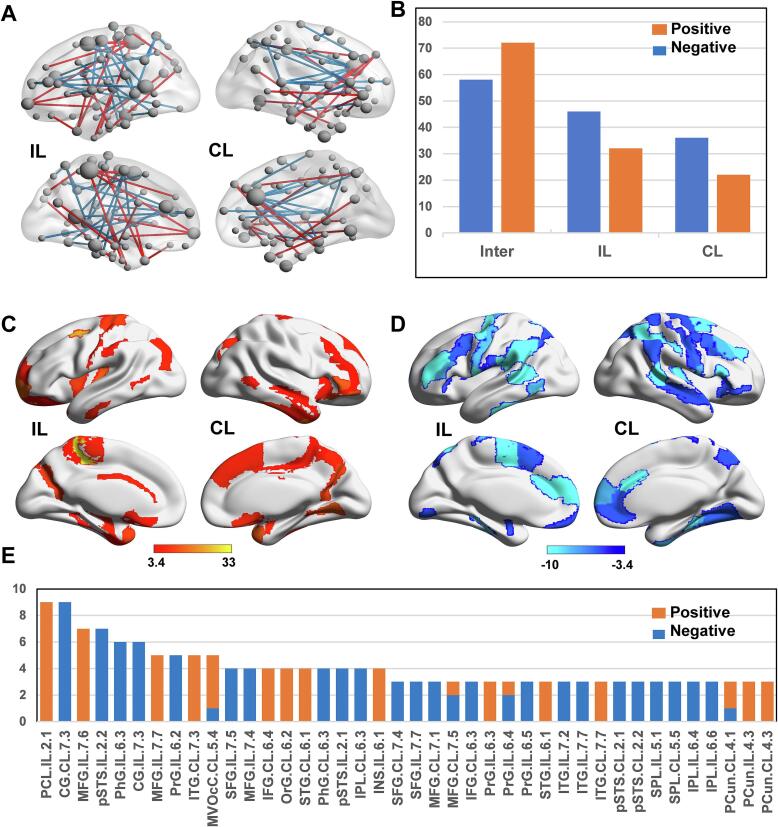
**Aberrant IDSCN connections in chronic stroke patients revealed by network-based statistics.** NBS identified 133 abnormal IDSCN connections in stroke patients (p < 0.05, FWE correction) (A), which mainly connect bilateral hemispheres (B). (C) and (D) visualize the areas distribution for positive and negative IDSCNs, respectively, and (E) shows the number of abnormal IDSCN connections of the top 40 areas. Abbreviations: FWE = family-wise error, IDSCN = individualized differential structural covariance network, NBS = network-based statistics, IL = ipsilesional, CL = contralesional.

NBS identified 133 connections in chronic stroke patients whose average IDSCN significantly deviated from the HC-derived norms (p < 0.05, FWE correction) (Fig. 2**A**), primarily linking bilateral hemispheres (36 positive and 29 negative connections), followed by within ipsilesional connections (16 positive and 23 negative connections) and contralesional connections (11 positive and 18 negative connections) (Fig. 2**B**). These aberrant IDSCN connections included 70 negative IDSCN connections involving 60 cerebral areas (Fig. 2**C**), and 63 positive IDSCN connections involving 61 cerebral areas (Fig. 2**D**), respectively. Furthermore, positive IDSCN connections predominantly targeted in ipsilesional (IL) paracentral lobes (IL.PCL-2.1 = 9 connections), IL middle frontal gyri (IL.MFG-7.6 = 7, IL.MFG-7.7 = 5), and contralesional (CL) inferior temporal gyrus (CL.ITG-7.3 = 5). In contrast, negative IDSCN connections primarily targeted bilateral cingulated gyri (IL CG-7.3 = 9, CL CG-7.3 = 6), IL posterior superior temporal sulcus (IL.pSTS-2.2 = 7), IL parahippocampal gyrus (IL.phG-6.3 = 6), and IL precentral gyrus (IL.PrG-6.2 = 5) (Fig. 2**E**).

### 4.3 Subnetwork distributions of the aberrant IDSCN

**Fig. 3:**
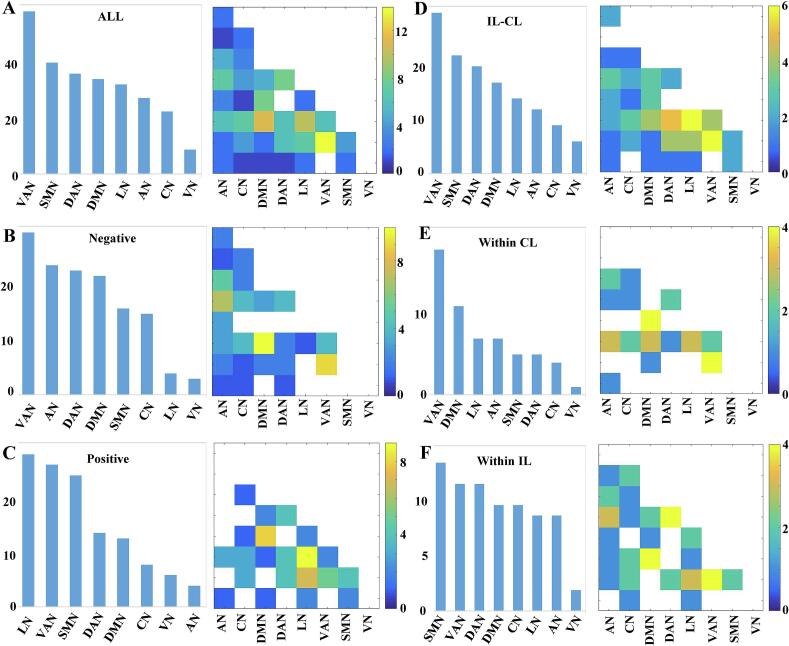
**Subnetwork distributions of the aberrant IDSCN Connections in chronic stroke patients.** Intra- and inter-networks among inter-hemisphere (**A**, **D**), ipsilesional (**B**, **E**), and contralesional (**C**, **F**). Significantly connections after NBS: **A**, **B**, **C**. Abbreviations: IDSCN = individualized differential structural covariance network, NBS = network-based statistics, IL = ipsilesional, CL = contralesional, AN = Auditory Network, CN = Control Network, DMN = Default Mode Network, DAN = Dorsal Attention Network, LN = Limbic Network, SMN = Somatic Motor Network, VAN = Ventral Attention Network.

We further categorized the deviated IDSCN connections into eight subnets. Among the 133 abnormal connections identified by NBS, we found that VAN exhibited the most significant number of abnormal IDSCN connections among stroke patients, followed by SMN and DAN (Fig. 3**A**). When examining the directionality of the effects, the top three negative IDSCN connections were observed in the VAN, AN, and DAN (Fig. 3**B**). Conversely, the top three positive IDSCN subnetworks included the LN, VAN, and SMN **(**Fig. 3**C)**. Regarding hemisphere-specific abnormalities, the top 3 subnetworks with abnormal inter-hemisphere IDSCN included the VAN, SMN, and DAN **(**Fig. 3**D**); in contrast, abnormal IDSCN predominantly affected contralesional VAN DMN and LN (Fig. 3**E**) and ipsilesional SMN, VAN, and DAN (Fig. 3**F**).

### 4.4 Regional GMV change and association with aberrant IDSCN

**Fig. 4:**
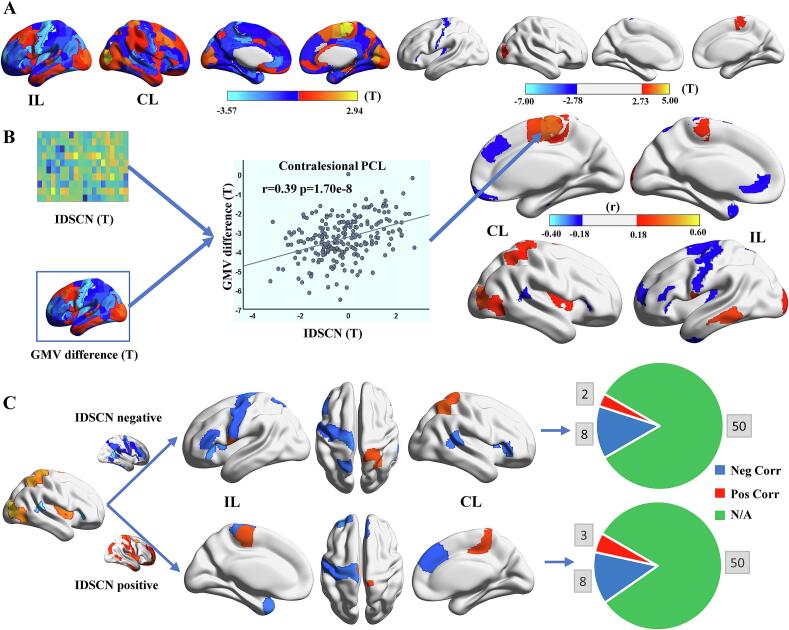
**Regional GMV change and association with aberrant IDSCN in chronic stroke patients. (A)** GMV differences between stroke patients and HC (left, un-threshold; right, p < 0.05 with FWE correction). **(B)** Associations between GMV changes and the IDSCN deviations (T values) in stroke patients. **(C)** Summary of associations in two types of aberrant IDSCN (negative vs positive deviations). Abbreviations: GMV = grey matter volume, FWE = family-wise error, IDSCN = individualized differential structural covariance network, IL = ipsilesional, CL = contralesional, corr = correlation.

Two-sample *t*-test identified five brain regions with atrophied GMV in stroke patients, including the ipsilesional PrG (2 ROIs), postcentral gyrus (PoG), opercular part of the inferior frontal gyrus (IFG), and posterior insular gyrus. Additionally, two regions exhibited increased GMV, namely the contralesional PCL and middle occipital gyrus (MOG) (p < 0.05, FWE correction). Notably, 5/7 regions are within the SMN subnetwork, and the remaining 2 are attributed to VAN and VN, respectively (Fig. 4**A**).

Spearman spatial correlation identified 35 brain regions where the IDSCNs in stroke patients were significantly associated with the regional GMV changes (p < 0.05, FDR correction), including 14 negative and 5 positive associations in the ipsilesional hemisphere, and 5 negative and 11 positive associations in the contralesional hemisphere (Fig. 4**B**). Notably, of all the brain regions (121) with significantly aberrant IDSCN, only a small portion(**21**) exhibited a significant association with GMV changes (Fig. 4**C**).

### 4.5 Association between aberrant IDSCN and clinical outcomes

**Fig. 5:**
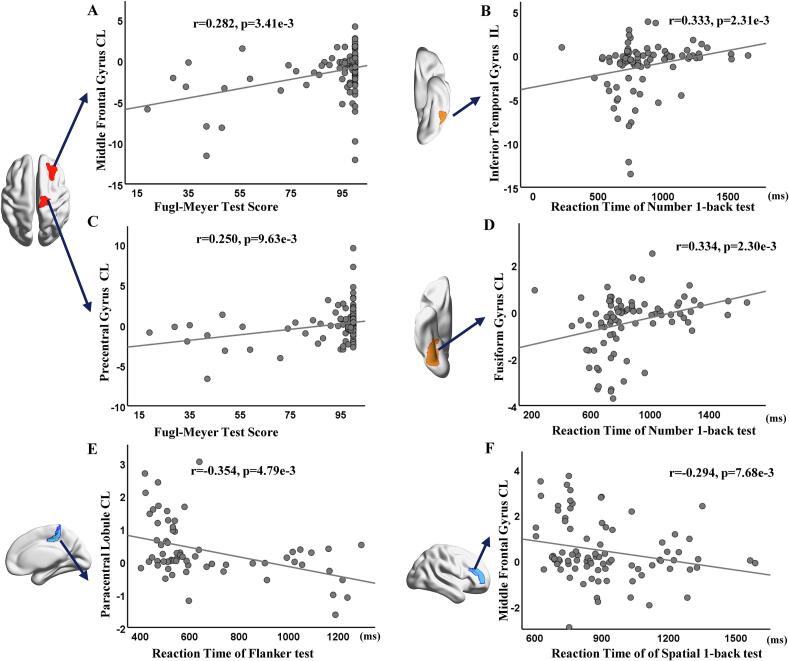
**Association between aberrant IDSCN and clinical outcomes in chronic stroke patients.** This figure only visualized association pairs of significant partial correlation coefficients with p < 0.01. For full associations, please see **Supplementary.**

Partial correlation analysis did not reveal a significant correlation between the abnormal IDSCN and clinical outcomes under FDR corrections (P < 0.05). We only found weak associations using an uncorrected threshold of p < 0.01 (Fig. 5). For instance, the FMT of chronic stroke showed a significantly positive correlation with the aberrant IDSCNs of contralesional MFG (r = 0.282, p = 3.41e-3) (Fig. 5**A)** and PrG (r = 0.250, p = 9.63e-3) (Fig. 5**C**). The reaction time of the number 1-back test exhibited a positive relationship with ipsilesional ITG (r = 0.333, p = 2.31e-3) (Fig. 5**B**) and contralesional fusiform gyrus (FuG) (r = 0.334, p = 2.30e-3) (Fig. 5**D**). The reaction time of the Flanker task demonstrated a negative relationship with contralesional PCL (r = -0.354, p = 4.78e-3) (Fig. 5**E**). Additionally, the reaction time of spatial 1-back task had a negative correlation with the contralesional MFG (r = -0.294, p = 7.68e-3) (Fig. 5**F**).

## 5 Discussion

This study explored the reorganization of the IDSCN in patients with chronic subcortical stroke. We observed a balanced increase and decrease in IDSCN after chronic stroke, particularly impacting interhemispheric and contralesional connections of VAN/SN, and ipsilesional connections of SMN. Additionally, we found that IDSCN could identify more reorganized brain regions than local GMV and exhibited weak and complex correlations with local GMV findings. Finally, abnormal IDSCN can imply long-term clinical recovery levels in motor skills, executive functions, and working memory. These findings suggest that IDSCN could offer additional insights into cortical reorganization and functional rehabilitation beyond regional morphometric measures.

The reorganization patterns of IDSCN following chronic subcortical stroke present a compelling illustration of the brain's adaptive reorganization capabilities. Notably, we found that interhemispheric connections are the most significantly affected, showing a balanced distribution of both increased and decreased IDSCN, which aligns with the adaptive reorganization of the functional network in stroke patients (Wang et al., 2010, Yuan et al., 2020, Brancaccio et al., 2022, Hensel et al., 2023, Liu et al., 2015). Furthermore, the VAN/SN emerges as the subnetwork where IDSCN reorganization is most pronounced, underscoring its pivotal role in recovery and adaptation processes post-stroke (Mattos et al., 2023, Brownsett et al., 2014). Additionally, the SMN and the DAN also primarily exhibited IDSCN alterations, suggesting a widespread impact across multiple cognitive and motor control networks after subcortical stroke (Griffis et al., 2019, Wang et al., 2014). The patterns of IDSCN reorganization within the ipsilesional and contralesional hemispheres are equally significant but differ in their respective subnetworks. The SMN exhibits the most substantial impact in the ipsilesional hemisphere, indicating a direct disruption (Fan et al., 2013, Liu et al., 2022); and subsequent within the ipsilesional (Favre et al., 2014) and interhemispheric (Zhang et al., 2014) reorganizations in regions critical for motor control and sensory processing. Conversely, the VAN/SN is more significantly impacted in the contralesional hemisphere, which may reflect compensatory adaptations of this subnetwork related to multi-dimensional functional rehabilitation, including motor, attention, and language, and more (Mattos et al., 2023, Brownsett et al., 2014, Corbetta and Shulman, 2011). These findings underscore the complexity of neural adaptations following a stroke and highlight the importance of considering both interhemispheric and intrahemispheric changes when developing therapeutic approaches.

On the association between IDSCN and regional GMV impairment in chronic subcortical stroke, we found that brain regions with altered IDSCN were significantly more extensive than those with regional GMV changes, consistent with previous studies (Wang et al., 2019, Wei et al., 2020, Veldsman et al., 2020). This finding suggests that the impact of subcortical stroke extends beyond the lesion site and affects not only the remote regions but also more widespread structural networks across the brain. Furthermore, we found regional GMV changes can account for only a small proportion of the IDSCN changes, implying that regional GMV alterations alone may not fully capture the complexity of brain structural reorganization following subcortical stroke, in contrast, IDSCN and other network-based metrics could provide additional insights to bridge this gap (Wang et al., 2019, Wei et al., 2020, Veldsman et al., 2020). Finally, our study underscores the intricate nature of the relationships between regional GMV and IDSCN reorganizations which can exhibit both positive and negative correlations simultaneously, even within a single brain region (such as the ipsilesional SMN), indicating that the impact of subcortical stroke on brain regional and network reorganization is not uniform, and may involve a delicate balance of compensatory and maladaptive processes (Xu et al., 2022, Siegel et al., 2016).

In this study, no significant correlation was found between inter-patient variability in IDSCN and functional recovery after applying multiple comparison corrections. Instead, we observed only weak, uncorrected evidence of an association. Several factors may explain the absence or weakness of this association: (1) the recruited patients generally demonstrated good functional recovery and mild symptoms at the chronic stage, resulting in limited clinical heterogeneity across patients, which may have weakened or obscured the association with IDSCN; (2) not all recruited patients underwent every dimension of clinical assessment, leading to relatively small sample sizes for each correlation analysis and, consequently, reduced statistical power. Therefore, the potential of IDSCN as a prognostic indicator and a target for rehabilitation should be further evaluated in future studies.

This study has several limitations. First, the recruited patients generally exhibited good functional recovery and mild symptoms at the chronic stage. This selection bias primarily arose from the retrospective recruitment of stroke patients. Severe patients often face challenges undergoing MRI examinations and completing clinical assessments on an outpatient basis. Consequently, this issue may underrepresent patients with poor outcomes, potentially limiting the generalization of our findings. Moreover, this issue could also account for the relatively weak association between IDSCN reorganization and functional disability/recovery, which requires validation in patients with greater symptom heterogeneities symptoms. Second, this is a cross-sectional study, which inherently limits the ability to track the dynamic changes of the IDSCN over time. Longitudinal studies are essential for observing the progression of IDSCN reorganization and for establishing more definitive causal relationships between IDSCN alterations and clinical outcomes in stroke patients. Third, IDSCN provides information solely about structural covariation between brain regions. Its relationship with other stroke-related structural and functional connectivity changes remains to be explored. Future studies should investigate the interplay between IDSCN and other brain networks to gain a more comprehensive understanding of post-stroke recovery. Finally, this study involves chronic stroke patients who are undergoing natural rehabilitation and have received various therapeutic interventions. However, the current sample size is insufficient to explore the impact of different rehabilitation methods on IDSCN remodeling and symptom recovery.

## CRediT authorship contribution statement

**Hongchuan Zhang:** Writing – review & editing, Writing – original draft, Validation, Software, Methodology, Investigation, Formal analysis, Data curation. **Jun Guo:** Funding acquisition, Data curation. **Jingchun Liu:** Resources, Project administration. **Caihong Wang:** Resources, Project administration. **Hao Ding:** Software, Methodology. **Tong Han:** Validation, Formal analysis. **Jingliang Chen:** Writing – review & editing, Supervision. **Chunshui Yu:** Writing – review & editing, Supervision. **Wen Qin:** Writing – review & editing, Validation, Supervision, Software, Methodology, Investigation, Funding acquisition, Conceptualization.

## Declaration of competing interest

The authors declare that they have no known competing financial interests or personal relationships that could have appeared to influence the work reported in this paper.

## Data Availability

Data will be made available on request.
