# Supplementary material for: Reorganization of cortical individualized differential structural covariance network is associated with regional morphometric changes in chronic subcortical stroke
Source: Neuroimage Clin. 2025 Jan 13;45:103735. doi: 10.1016/j.nicl.2025.103735 (PMC11787593; doi:10.1016/j.nicl.2025.103735)
Supplement: Supplementary Data 1 [file mmc1.docx]

Reorganization of cortical individualized differential structural covariance network is associated with regional morphometric changes and functional recovery in chronic subcortical stroke

# Supplementary Methods

## Measurement of motor and cognitive functions

### Fugl-Meyer Test (FMT)

FMT in the current study refers to whole extremities which comprise the upper and lower extremities with a total of 66 scores and 34 scores respectively. The assessment of the 33 items about upper extremities involves a meticulous evaluation, with each item classified into three distinct categories: 0 (no movement),1 (partial movement), and 2 (free movement). Consequently, the scores span a range from 0 to 66. The lower extremities have 17 items similar to the assessment of upper extremities, with scores ranging from 0 to 34. While the total score of FMT in the current study was 100. FMT is a widely used quantitative measure for assessing motor function, balance, sensation, and joint functioning in patients who have had a stroke. It provides a comprehensive evaluation of motor recovery.

### Flanker Test

A modified version of the Attention Network Test (ANT) was used to assess attention function. E-prime software 2.0 (Psychology Software Tools, Pittsburgh, PA, USA) was used for visual stimulus presentation and response recording. Participants were required to respond to the direction of a central arrow (target) and to ignore adjacent congruent or incongruent distracting arrows by pressing a button on the computer with their non-paretic hand. The software measures both reaction time (F_RT) and accuracy (F_ACC), providing insights into the participants' executive function and cognitive control.

**Spatial 1-back Test**

In E-Prime software, a spatial 1-back task is typically designed to assess working memory by requiring participants to monitor a sequence of spatial locations and respond when a location matches the one presented immediately before it. First, five arrows point either left or right, displayed in three rows: the middle row contains three arrows, while one arrow is shown in each of the rows above and below. A target appears in one of these locations for 0.5s duration. A blank screen or fixation cross is shown for 3.5s between stimuli. Participants are instructed to press a specific key if the current location matches the previous one as fast as possible (1-back match), No response if the array does not match. Reaction times (S_RT) and accuracy for each trial (S_ACC) are recorded representing the participant’s spatial memory.

**Number 1-back Test:** Like spatial 1-back test, a target array is replaced by a fixed number. The timing between stimuli mimics the test by Spatial 1-back test. Reaction time (N_RT) and accuracy (N_ACC) are recorded reflecting participants' ability to maintain and update numerical information in working memory.

# Supplementary Figures


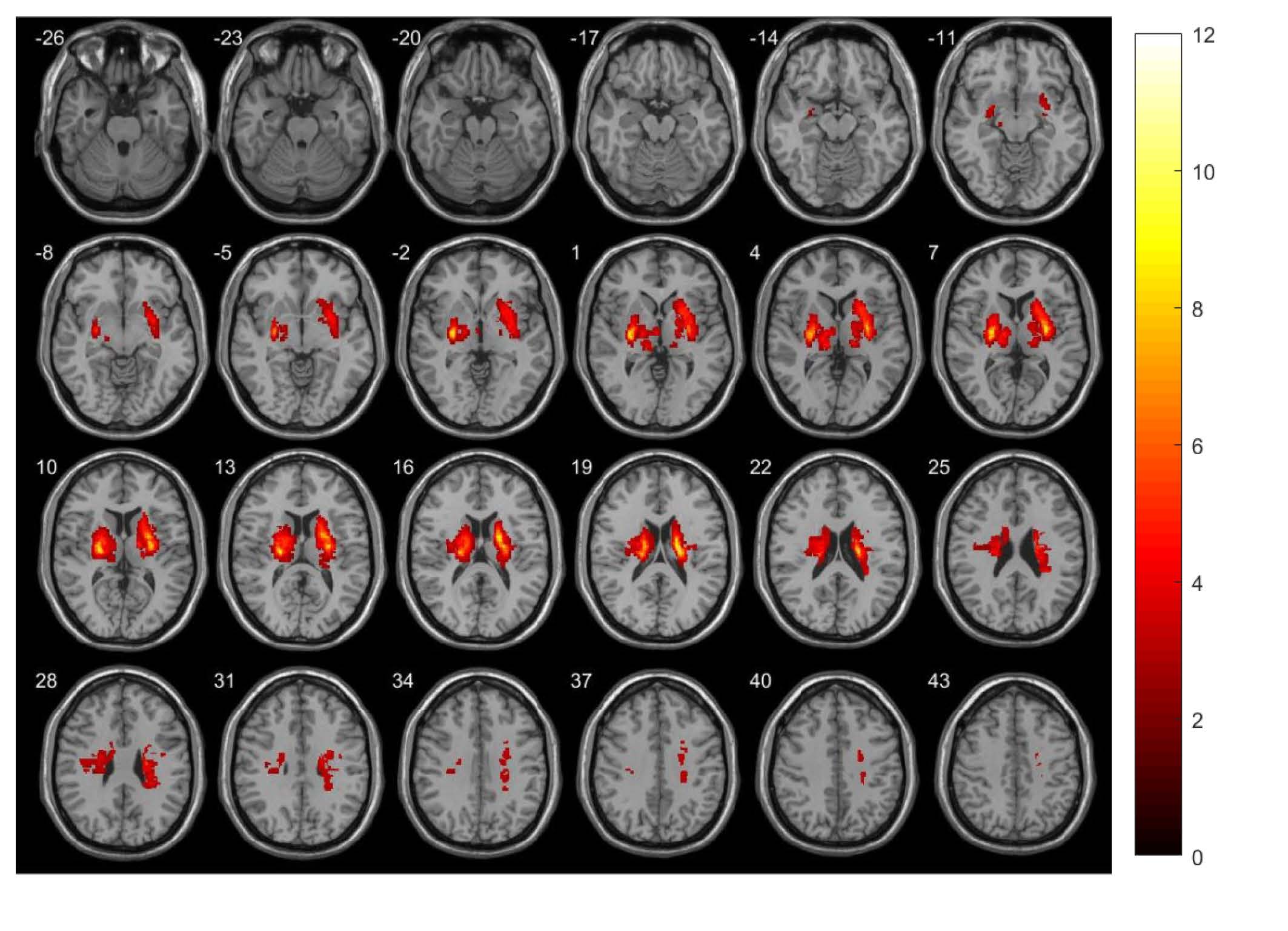


### **Supplementary Figure S1. Lesion incidence map of patients with subcortical stroke**.

**
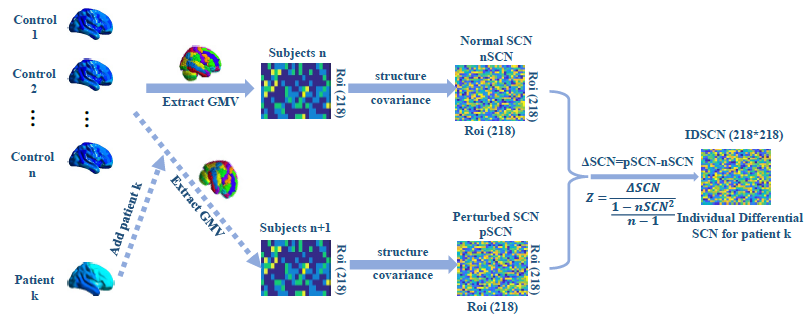
**

Supplementary Figure S2. Workflow for constructing IDSCN**.** Firstly, a reference group-wise structural covariance network (SCN) was established comprising all (n) healthy controls for each site (referred to as nSCN). Subsequently, a patient (k) was introduced to the health controls to construct a perturbed structural covariance network (pSCN). Then ΔSCN was calculated by subtracting nSCN from pSCN. Finally, Individualized Differential Structural Covariance Network (IDSCN) was calculated by Z transformation of the ΔSCN for patient k. Element in IDSCN denotes the edge connected two ROIs in patient k deviates from the healthy normal in that site. Abbreviations: IDSCN = individualized differential structural covariance network.

# Supplementary Tables

### **Supplementary Table S1: Demographic and clinical information for involved participant**s

| **Site** |  | **Age** | **Gender** | **Lesion** | **FMT** | **F_RT** | **F_ACC** | **N_RT** | **N_ACC** | **S_RT** | **S_ACC** |
| --- | --- | --- | --- | --- | --- | --- | --- | --- | --- | --- | --- |
|  |  | (year) | (M/F) | left/right |  | ms |  | ms |  | ms |  |
| **TJHH** | Stroke | 56.6±6.4 | 18/4 | 10/12 | 100(73,100) | - | - | 780(719,917) | 0.930(0.825,0.970) | 836(761,882) | 0.890(0.850,0.930) |
|  | HC | 56.1±6.8 | 13/10 | - | 100(100,100) | 528(485,571) | 1.000(0.980,1.000) | 771(673,838) | 0.890(0.850,0.950) | 776(707,868) | 0.940(0.890,0.950) |
|  | *P* value | 0.460 | 0.070 | - | 3.33e-4* | - | - | 0.368 | 0.399 | 0.306 | 3.40e-2* |
| **FAHZU** | Stroke | 53.8±8.6 | 27/9 | 22/14 | 100(96,100) | 591(516,1053) | 0.983(0.967,1.000) | 994(737,1224) | 0.959(0.908,0.984) | 934(763,1241) | 0.932(0.872,0.967) |
|  | HC | 53.0±8.0 | 21/14 | - | 100(100,100) | 565(465,878) | 0.983(0.967,1.000) | 907(697,1070) | 0.951(0.902,0.980) | 922 (713,1019) | 0.930(0.900,0.951) |
|  | *P* value | 0.063 | 0.170 | - | 5.00e-3* | 0.171 | 0.537 | 0.178 | 0.755 | 0.105 | 0.556 |
| **TMUGH1** | Stroke | 57.2±7.7 | 20/11 | 16/15 | 100(96,100) | 535(487,590) | 0.980(0.930,1.000) | 774(717,811) | 0.920(0.860,0.950) | 821(743,966) | 0.920(0.870,0.950) |
|  | HC | 55.9±7.7 | 26/13 | - | 100(100,100) | 523(485,560) | 0.980(0.955,1.000) | 694(635.781) | 0.930(0.905,0.950) | 746(667,840) | 0.920(0.870,0.950) |
|  | *P* value | 0.400 | 0.850 | - | 3.10e-5* | 0.290 | 0.276 | 0.139 | 0.331 | 3.40e-2* | 0.888 |
| **TMUGH2** | Stroke | 56.5±7.6 | 16/7 | 9/14 | - | - | - | - | - | - | - |
|  | HC | 56.8±6.9 | 14/11 | - | - | - | - | - | - | - | - |
|  | *P* value | 0.710 | 0.330 | - | - | - | - | - | - | - | - |

**Abbreviations:** TJHH=Tianjin HuanHu Hospital; FAHZU=The First Affiliated Hospital of Zhengzhou University; TMUGH1=Tianjin Medical University General Hospital Dataset 1; TMUGH2=Tianjin Medical University General Hospital Dataset 2; FMT=Fugl-Meyer Test total scores; F_RT=Reaction Time of Franker test; F_ACC=Accuracy of Franker test; N_RT=Reaction Time of Number 1-back test; N_ACC= Accuracy of Number 1-back test; S_RT= Reaction Time of Spatial 1-back test; S_ACC= Accuracy of Spatial 1-back test.

### Supplementary Table S2. MRI scanning information for each dataset.

| **Dataset** | **TJHH** | **FAHZU** | **TMUGH1** | **TMUGH2** |
| --- | --- | --- | --- | --- |
| Scanner Model | SIEMENS TrioTim | GE MR750 | GE MR750 | GE HDxt |
| Sequence | MPRAGE | BRAVO | BRAVO | BRAVO |
| TR (ms) | 2000 | 8.2 | 8.2 | 8.1 |
| TE (ms) | 2.3 | 3.2 | 3.2 | 3.1 |
| TI (ms) | 900 | 450 | 450 | 450 |
| FA (°) | 9 | 11 | 11 | 13 |
| Slices | 192 | 188 | 188 | 176 |
| Matrix | 256 x 232 | 256 x 256 | 256 x 256 | 256 x 256 |
| Voxel size(mm^3^) | 1 x 1 x 1 | 1 x 1 x 1 | 1 x 1 x 1 | 1 x 1 x 1 |

Abbreviations: BRAVO=brain volume; FA=flip angle; MPRAGE=magnetization prepared rapid acquisition gradient echo; TE=echo time; TI=inversion time; TR=repetition time.

**Supplementary Table S3. The correlation between  IDSCN and clinic assessments.**

| **ROI** | **FMT_ALL** | | **F_RT** | | **N_RT** | | **S_RT** | | **F_ACC** | | **N_ACC** | | **S_ACC** | |
| --- | --- | --- | --- | --- | --- | --- | --- | --- | --- | --- | --- | --- | --- | --- |
|  | **P** | **r** | **P** | **r** | **P** | **r** | **P** | **r** | **P** | **r** | **P** | **r** | **P** | **r** |
| **SFG.IL.7.1** | 0.513 | 0.064 | 0.930 | -0.011 | ***0.047*** | ***-0.221*** | 0.825 | -0.025 | 0.442 | -0.099 | 0.566 | 0.065 | 0.398 | -0.096 |
| **SFG.CL.7.3** | ***0.046*** | ***0.195*** | 0.852 | -0.024 | 0.563 | -0.065 | 0.597 | -0.060 | 0.568 | 0.074 | 0.212 | -0.140 | 0.313 | 0.114 |
| **SFG.CL.7.4** | 0.615 | 0.049 | 0.141 | 0.189 | 0.722 | 0.040 | 0.576 | 0.063 | 0.158 | -0.182 | ***0.023*** | ***-0.253*** | 0.984 | 0.002 |
| **SFG.IL.7.5** | 0.490 | -0.068 | 0.297 | 0.135 | 0.242 | 0.131 | 0.258 | 0.127 | 0.497 | 0.088 | ***0.032*** | ***0.239*** | 0.081 | 0.196 |
| **MFG.CL.7.1** | ***0.010*** | ***0.250*** | 0.658 | -0.057 | 0.833 | 0.024 | 0.799 | -0.029 | 0.179 | -0.173 | 0.535 | 0.070 | 0.540 | -0.070 |
| **MFG.IL.7.2** | 0.185 | 0.130 | 0.271 | 0.142 | ***0.011*** | ***0.280*** | 0.077 | 0.198 | 0.695 | 0.051 | 0.689 | -0.045 | 0.632 | -0.054 |
| **MFG.IL.7.4** | ***0.044*** | ***0.196*** | 0.660 | -0.057 | 0.430 | 0.089 | 0.587 | -0.061 | 0.095 | -0.214 | 0.643 | -0.052 | 0.136 | -0.168 |
| **MFG.CL.7.4** | 0.555 | 0.058 | 0.413 | -0.106 | 0.107 | -0.181 | ***0.008*** | ***-0.294*** | 0.648 | 0.059 | 0.990 | 0.001 | 0.076 | -0.199 |
| **MFG.IL.7.6** | ***0.011*** | ***0.245*** | 0.354 | -0.120 | 0.643 | 0.052 | 0.556 | -0.066 | 0.728 | 0.045 | 0.563 | 0.065 | 0.599 | -0.060 |
| **MFG.IL.7.7** | 0.655 | -0.044 | 0.068 | 0.233 | ***0.026*** | ***0.247*** | 0.058 | 0.212 | 0.753 | 0.041 | 0.296 | 0.117 | 0.919 | 0.012 |
| **PrG.IL.6.1** | ***0.021*** | ***0.224*** | ***0.029*** | ***-0.278*** | 0.256 | -0.128 | 0.062 | -0.208 | 0.997 | 0.000 | 0.586 | 0.061 | 0.414 | -0.093 |
| **PrG.CL.6.1** | 0.686 | -0.040 | 0.524 | -0.083 | 0.595 | -0.060 | 0.570 | -0.064 | 0.365 | -0.117 | 0.206 | -0.142 | ***0.027*** | ***-0.248*** |
| **PrG.CL.6.2** | 0.509 | 0.065 | ***0.039*** | ***0.263*** | ***0.034*** | ***0.236*** | ***0.026*** | ***0.248*** | 0.330 | 0.126 | 0.811 | -0.027 | 0.292 | -0.119 |
| **PrG.CL.6.4** | ***0.003*** | ***0.282*** | 0.185 | -0.170 | 0.075 | -0.199 | 0.094 | -0.188 | 0.773 | -0.037 | 0.606 | 0.058 | 0.440 | -0.088 |
| **PCL.IL.2.1** | 0.539 | -0.060 | 0.867 | 0.022 | 0.866 | -0.019 | 0.440 | -0.087 | 0.857 | 0.023 | 0.448 | -0.086 | ***0.040*** | ***-0.230*** |
| **PCL.CL.2.1** | 0.861 | 0.017 | ***0.005*** | ***-0.354*** | ***0.025*** | ***-0.249*** | 0.191 | -0.147 | 0.730 | -0.045 | 0.161 | 0.157 | 0.504 | 0.076 |
| **PCL.IL.2.2** | ***0.033*** | ***0.207*** | 0.346 | -0.122 | 0.176 | -0.152 | 0.278 | -0.122 | 0.877 | -0.020 | 0.422 | 0.090 | 0.334 | -0.109 |
| **MTG.IL.4.3** | 0.129 | 0.148 | ***0.016*** | ***-0.304*** | 0.113 | -0.177 | 0.088 | -0.191 | ***0.014*** | ***-0.310*** | 0.605 | -0.058 | 0.703 | -0.043 |
| **ITG.IL.7.2** | 0.139 | 0.145 | 0.119 | 0.200 | ***0.002*** | ***0.334*** | 0.238 | 0.133 | 0.076 | 0.227 | 0.814 | 0.027 | 0.522 | 0.073 |
| **ITG.CL.7.3** | ***0.017*** | ***0.231*** | 0.966 | 0.006 | 0.939 | 0.009 | 0.841 | 0.023 | 0.811 | -0.031 | 0.983 | 0.002 | 0.119 | 0.176 |
| **ITG.CL.7.7** | ***0.039*** | ***0.200*** | 0.416 | 0.105 | 0.921 | 0.011 | 0.325 | 0.111 | 0.292 | 0.136 | 0.575 | 0.063 | 0.116 | 0.177 |
| **FuG.CL.3.2** | 0.731 | 0.034 | 0.113 | 0.204 | ***0.002*** | ***0.334*** | 0.163 | 0.156 | 0.191 | 0.168 | 0.616 | -0.057 | 0.779 | 0.032 |
| **PhG.IL.6.3** | 0.465 | 0.072 | 0.685 | 0.052 | 0.656 | -0.050 | 0.226 | -0.136 | 0.920 | -0.013 | 0.690 | 0.045 | ***0.048*** | ***0.222*** |
| **pSTS.CL.2.1** | 0.999 | 0.000 | 0.304 | -0.133 | 0.882 | 0.017 | 0.492 | 0.077 | ***0.037*** | ***-0.266*** | 0.457 | -0.084 | 0.213 | -0.141 |
| **IPL.IL.6.1** | 0.281 | 0.106 | ***0.018*** | ***-0.300*** | 0.755 | -0.035 | 0.710 | -0.042 | 0.086 | -0.220 | 0.750 | 0.036 | 0.641 | -0.053 |
| **IPL.CL.6.4** | 0.326 | 0.096 | 0.730 | 0.045 | 0.922 | 0.011 | 0.252 | 0.129 | 0.560 | -0.075 | 0.283 | 0.121 | ***0.020*** | ***0.259*** |
| **PCun.IL.4.3** | 0.284 | 0.105 | ***0.039*** | ***-0.263*** | 0.512 | -0.074 | 0.428 | -0.089 | 0.583 | 0.071 | 0.929 | -0.010 | 0.385 | 0.098 |
| **PCun.CL.4.4** | ***0.040*** | ***0.200*** | 0.123 | -0.198 | 0.219 | -0.138 | 0.104 | -0.182 | 0.719 | -0.047 | 0.784 | -0.031 | 0.299 | -0.118 |
| **PoG.CL.4.1** | 0.393 | -0.084 | 0.086 | -0.220 | 0.787 | 0.031 | 0.757 | -0.035 | 0.894 | -0.017 | ***0.049*** | ***-0.219*** | 0.277 | -0.123 |
| **PoG.IL.4.2** | 0.169 | 0.135 | 0.832 | -0.027 | 0.296 | -0.118 | 0.487 | -0.078 | ***0.030*** | ***0.276*** | 0.146 | 0.163 | 0.674 | 0.048 |
| **INS.IL.6.4** | 0.371 | 0.088 | 0.146 | 0.187 | 0.099 | 0.184 | ***0.036*** | ***0.234*** | 0.498 | 0.088 | 0.124 | 0.172 | ***0.015*** | ***0.272*** |
| **CG.IL.7.2** | ***0.048*** | ***0.193*** | 0.633 | 0.062 | 0.125 | 0.172 | 0.166 | 0.156 | 0.404 | -0.108 | 0.760 | -0.034 | 0.220 | 0.139 |
| **CG.IL.7.3** | 0.874 | -0.016 | 0.645 | -0.060 | 0.708 | 0.042 | 0.844 | -0.022 | 0.865 | 0.022 | 0.181 | 0.150 | ***0.013*** | ***0.275*** |
| **CG.CL.7.5** | 0.415 | 0.080 | ***0.043*** | ***0.258*** | 0.104 | 0.182 | 0.134 | 0.168 | 0.400 | -0.109 | 0.437 | -0.088 | 0.876 | -0.018 |
| **CG.CL.7.6** | 0.205 | -0.124 | 0.815 | -0.030 | 0.425 | -0.090 | 0.722 | -0.040 | 0.530 | 0.081 | ***0.049*** | ***0.220*** | 0.418 | -0.092 |
| **Amyg.CL.2.2** | 0.285 | 0.105 | 0.296 | 0.135 | ***0.017*** | ***0.265*** | ***0.041*** | ***0.228*** | 0.696 | -0.051 | 0.640 | -0.053 | 0.178 | -0.152 |

**Note:** ***Black italic*** represents P < 0.05, with ***underline*** highlights p < 0.01.
